# Proliferative Sparganosis Due to *Spirometra mansoni* in a Dog: A Detailed Diagnostic Picture

**DOI:** 10.1002/vms3.71206

**Published:** 2026-08-29

**Authors:** Stephanie Middlemast, Nicholas Lim, Charles G. Gauci, Abdul Jabbar

**Affiliations:** ^1^ Greencross Vet Hospital University of Melbourne Werribee Victoria Australia; ^2^ Department of Veterinary Biosciences University of Melbourne Werribee Victoria Australia

**Keywords:** Australia, dog, parasites, proliferative sparganosis, Spirometra mansoni, ultrasound

## Abstract

A young Blue Heeler presented for abdominal distension and weight gain, without a history of raw feeding or travel outside the western suburbs of Melbourne, Victoria, Australia. Abdominal ultrasound noted innumerable small anechoic oval cystic structures scattered throughout the peritoneal cavity and suspended in a large volume of echogenic peritoneal effusion. Subsequent diagnostics, including computed tomography, exploratory laparotomy, histopathology and molecular testing, confirmed the structures were *Spirometra mansoni* plerocercoids, and the patient was diagnosed with proliferative sparganosis. Despite treatment, the animal's health rapidly declined and was euthanised three weeks later. The postmortem revealed extensive diffuse severe granulomatous peritonitis with fibrinous adhesions throughout the peritoneal cavity. Proliferative sparganosis is a rare and devastating parasitic condition resulting from asexual replication of *Spirometra* spp. larvae within body cavities or organs. This article provides a detailed diagnostic picture of canine peritoneal proliferative sparganosis and is the first report describing the ultrasonographic findings of this condition.

## Graphical abstract

Innumerable small oval cystic structures were identified in the peritoneal cavity of a young dog presenting with abdominal distension and weight gain. A combination of diagnostics confirmed these structures as *Spirometra mansoni* plerocercoids and established a diagnosis of proliferative sparganosis. This article provides a detailed diagnostic picture of this rare and devastating condition, including its previously unreported ultrasonographic appearance.

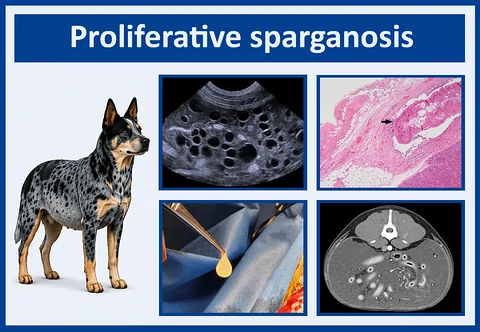

## 1 Introduction

Proliferative sparganosis is a rare zoonotic parasitic disease caused by the larval stages of the pseudophyllidean tapeworm, *Spirometra* (Vanathy 2022). The *Spirometra* life cycle begins when eggs are shed in the faeces of a definitive host (canids or felids) into freshwater, where they hatch into free‐swimming coracidia within approximately 1–2 weeks under suitable temperature conditions. The coracidia are ingested by freshwater copepods (first intermediate hosts), within which they develop into procercoid larvae over approximately 2–3 weeks. When infected copepods are consumed by a second intermediate or paratenic host, such as frogs, tadpoles, snakes, or small mammals, the procercoid migrates into the musculature or connective tissues and develops into the plerocercoid (sparganum) stage, which represents the infective stage for definitive hosts and incidental hosts, including humans and domestic animals. The duration of plerocercoid development in paratenic hosts is not precisely defined and may persist for the life of the host. Definitive hosts become infected by ingesting paratenic hosts harbouring spargana, after which the plerocercoid develops into an adult tapeworm in the small intestine, with patency reported to occur within approximately 2–3 weeks post‐infection (Vanathy 2022; Kuchta et al. 2024; Wu et al. 2024).

The adult *Spirometra* may cause mild clinical signs such as diarrhoea, vomiting and weight loss when present within the small intestine of a definitive host (CAPC 2024). Sparganosis, on the other hand, occurs when there is ingestion of the larval form, either through ingestion of the first intermediate host (contaminated water), ingestion of the second intermediate host, or direct transmission through open wounds, eyes and mucosa (Cui et al. 2017), whereby the larval form migrates and enters various host tissue or body cavities (Kuchta et al. 2024; Simpson et al. 2012). Hosts infected in this way are termed paratenic hosts, and while dogs and cats typically act as the definitive host (harbouring the adult worm), they can also rarely act as a paratenic host (harbouring the larval form). In paratenic hosts, the larval plerocercoids replicate asexually outside of the small intestine, and the subsequent condition is termed proliferative sparganosis. This term has historically only been attributed to *Sparganum* spp., a similar but genetically distinct tapeworm species (Miyadera et al. 2001; Kikuchi et al. 2021), however recent reports have documented cases of asexual replication due to *Spirometra* spp., (Miller et al. forthcoming Semenova et al. 2025; Tokiwa et al. 2024; Abdu and Sasaki 2026).

The distribution of *Spirometra* spp. is cosmopolitan, with an increased prevalence in warm and humid climates (Badri et al. 2022; Dybing et al. 2013). However, the occurrence of proliferative sparganosis is rare, with only six published articles describing proliferative sparganosis in dogs worldwide: two from Australia (Simpson et al. 2012; Beveridge et al. 1998), three from the USA (Miller et al. forthcoming, Semenova et al. 2025; Drake et al. 2008), and one from Germany (Stief and Enge 2011). In all cases, plerocercoid larvae were observed outside of the gastrointestinal tract, with severe granulomatous inflammation and fibrosis observed in the peritoneal (Miller et al. forthcoming; Semenova et al. 2025; Beveridge et al. 1998; Drake et al. 2008; Stief and Enge 2011) and thoracic cavities (Simpson et al. 2012). Due to the rarity of cases, a consensus on the recommended treatment has not been reached. In almost all cases, the patients were euthanised due to disease progression or a poor prognosis.

Humans can also be infected, typically as paratenic hosts (Tran et al. 2019). However, they can also rarely act as the definitive host (Kuchta et al. 2015; Lee et al. 1984), making sparganosis a zoonotic and public health concern (Badri et al. 2022). Most human infections lead to non‐proliferative sparganosis (rather than proliferative) and often result in localised subcutaneous nodules (Lu et al. 2014), with four reported cases in Australia dating back as early as 1905 (Tran et al. 2019). In these cases, surgical excision is the treatment of choice, and all native Australian cases occurred in a rural setting with possible exposures, including drinking contaminated, unfiltered or tank water or ingesting wild animals, including snakes, pigs, rabbits and fish.

This article presents a case report of peritoneal proliferative sparganosis in a young Australian dog. It provides a detailed diagnostic picture, including the findings of ultrasound, computed tomography, exploratory laparotomy and postmortem. To the authors’ knowledge, this is the first published case describing the ultrasonographic findings of peritoneal proliferative sparganosis in any domestic animal species. The article also aims to increase awareness of this zoonotic condition as a differential for abdominal distension in dogs.

## 2 Case Presentation

A 3‐year‐and‐9‐month‐old male neutered blue heeler was referred to Greencross Vet Hospital at the University of Melbourne, Werribee, Australia, for further diagnostics following a two‐week history of unintentional weight gain and noticeable abdominal distension following a change in diet. The animal was kept at an urban residence adjacent to the Cheetham Wetlands (37°55'05.0“S 144°47'25.6”E), Point Cook, Victoria, Australia, and had been fed a supermarket brand dry food. Weight was 26.8 kg on presentation, having gained 3.8 kg two weeks prior. The animal had been in the owner's possession since it was a puppy, living in the western suburbs of Melbourne, Australia, with no history of travel outside the local suburbs or to farmland, and without access to offal. Occasionally, frogs were observed in the animal's yard due to nearby wetlands, and seldom would it drink from puddles. Yearly vaccinations were up to date, but regular deworming was not undertaken. However, Nexgard Spectra (Afoxolaner, Milbemycin oxime, Boehringer Ingelheim Animal Health Australia Pty. Ltd, New South Wales) and Simparica Trio (Pyrantel embonate, Sarolaner, Moxidectin, Zoetis Australia, New South Wales) were used in the last 12 months. None of these products contained anticestodal drugs. The dog's energy, appetite, drinking and urination were normal, without a history of vomiting. However, diarrhoea was observed, and it was described as brown to orange and soft.

Physical examination revealed that the animal was bright, alert and responsive, with a normal body condition score and vitals. The abdomen was moderately distended and diffusely firm on palpation. No external wounds or external lymphadenopathy was observed, and the remainder of the physical examination was unremarkable.

## 3 Initial Diagnostics

### 3.1 Haematology and Biochemistry

Haematological and biochemical analyses revealed a mild eosinophilia (1.8 × 10^9^/L; Reference interval [RI]: 0.1‐1.3 × 10^9^/L), mild hypocholesterolaemia (3.1 mmol/L; RI: 3.9‐7.8 mmol/L), mildly increased amylase (1347 U/L; RI: 180–1200 U/L), mild hypoproteinaemia (50 g/L; RI: 51–72 g/L), borderline low normal albumin (31 g/L; RI: 31–44 g/L). Faecal examination using a standard centrifugal flotation technique and the Snap *Giardia* test (IDEXX Australia, New South Wales) revealed negative results.

### 3.2 Abdominal Ultrasound

**FIGURE 1:**
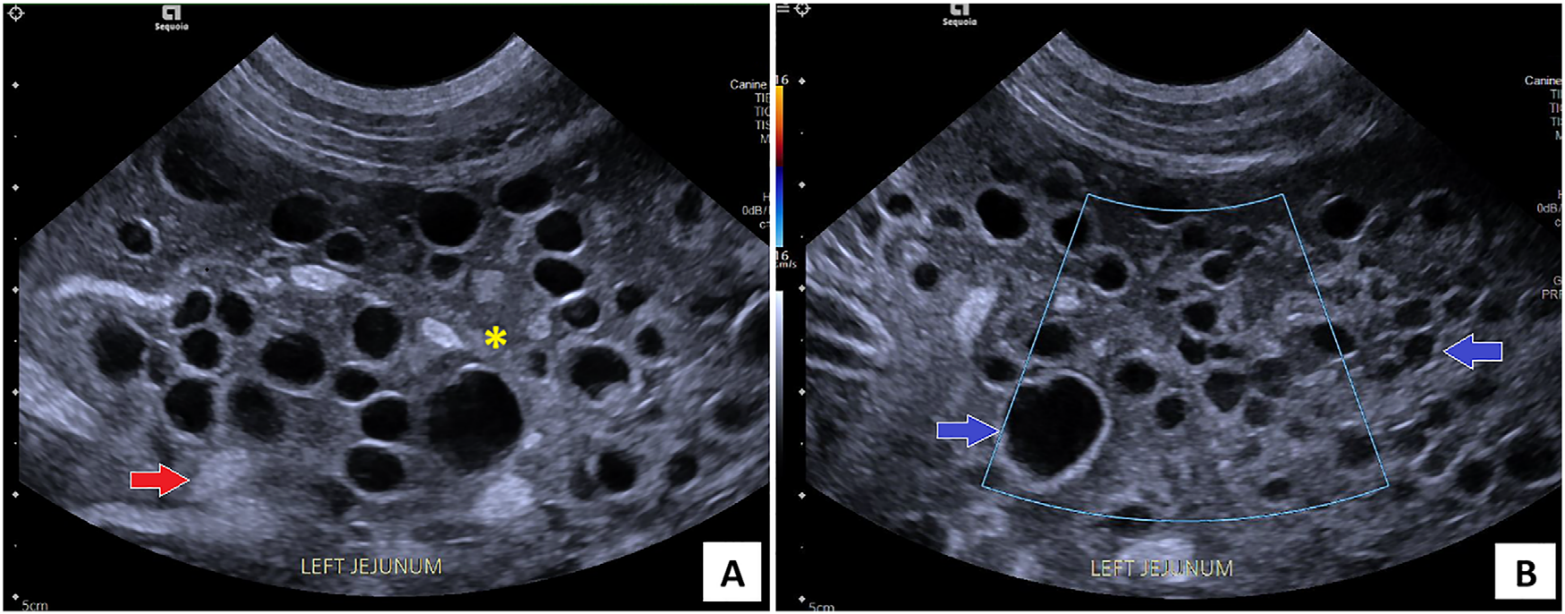
Ultrasound images showing innumerable variably sized avascular anechoic cysts (blue arrows) suspended in a large volume of peritoneal effusion (*) interlaced with strands of hyperechoic mesentery (red arrows).

An abdominal ultrasound was performed with an 11M3 Micro‐curved Array Transducer (Acuson Sequoia Ultrasound System, Siemens, Australia), which noted innumerable small anechoic oval cysts scattered throughout the peritoneal cavity adjacent to but not within organs. These cystic structures, measuring between 2 to 18 mm in diameter, with well‐defined, smooth, thin hyperechoic walls, were Doppler negative and suspended in a large volume of echogenic peritoneal effusion (Figure 1, Supplement Video 1). The surrounding mesentery was irregular and hyperechoic, with strands extending between the cysts and the small intestinal loops. The right medial iliac and the jejunal lymph nodes were mildly enlarged, with a height of 8.2 mm; RI: 4.1‐5.6 mm and 8.9 mm; RI: 5–7 mm, respectively (Ganesan et al. 2016). The colon walls were mildly thickened (2.6 mm; RI: 1.1‐1.9 mm (Gladwin et al. 2014)). The pancreas had irregular margination, hypoechoic echogenicity and a coarse echotexture, most consistent with mild pancreatitis.

Ultrasound‐guided abdominocentesis was performed, collecting 0.5 mL of serosanguinous fluid from between the cystic structures. Cytologic evaluation was consistent with cystic fluid with evidence of prior haemorrhage and no distinct infectious organisms. The bacterial culture was negative.

A prioritised differential list for the innumerable peritoneal cysts, with concurrent mild eosinophilia, included parasitic cysts (proliferative sparganosis caused by *Spirometra* sp.), least likely peritoneal larval cestodiasis (*Mesocestoides* spp. not reported in Australia), disseminated neoplasia (carcinomatosis, cystic leiomyomatosis, cystic lymphoma, with paraneoplastic eosinophilia) and chronic peritonitis. The lymph nodes were most likely reactive.

## 4 Further Diagnostics

The patient returned a week later, with the owner noticing mild lethargy and light brown diarrhoea. Additional weight gain of 300 g was noted, with unremarkable physical examination findings. The animal was admitted for a CT scan and exploratory laparotomy.

### 4.1 CT Scan

**FIGURE 2:**
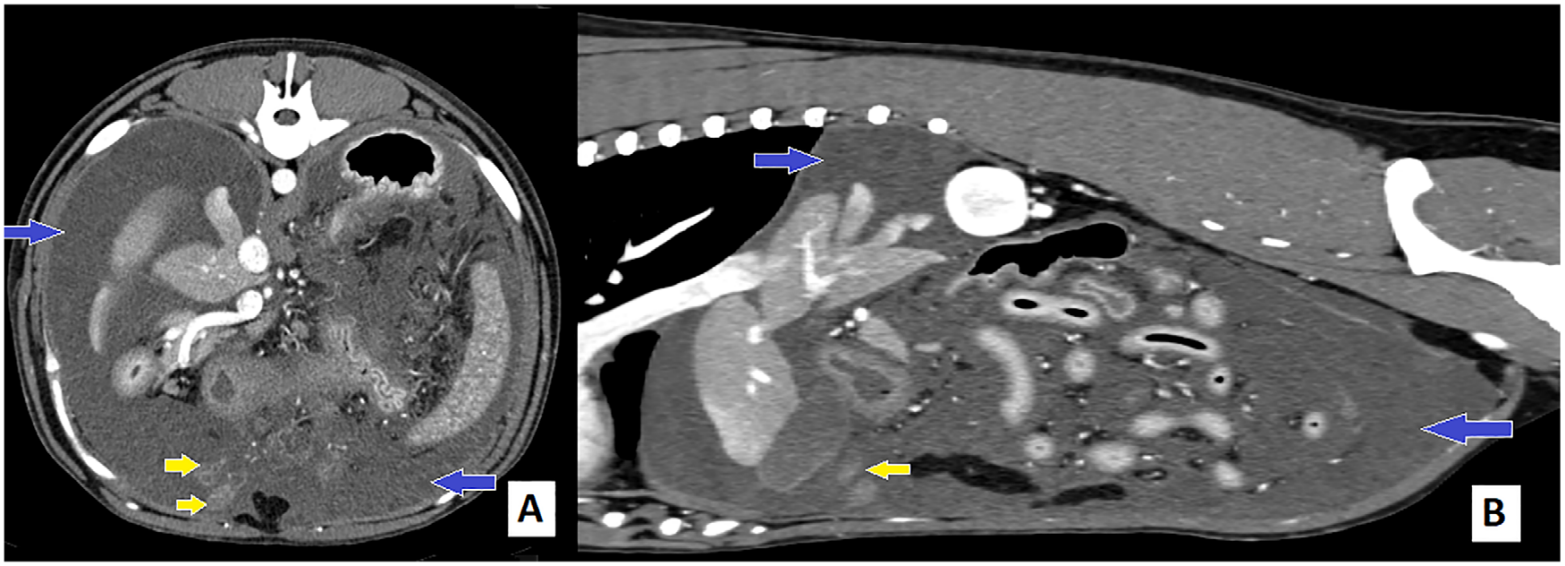
CT images in the transverse (A) and sagittal (B) planes showing marked peritoneal effusion (blue arrows) and two nodular lesions just caudal to the gallbladder (yellow arrows), compatible with parasitic peritonitis and granulomatous/cystic lesions.

The CT scan noted a severe abdominal effusion, two mildly irregular nodular lesions just caudal to the gall bladder (Figure 2), and one nodular thickening within the plica of the vena cava. The ventral margin of the liver had a mildly irregular to nodular thickening, and these changes were compatible with parasitic peritonitis with granulomatous/cystic lesions. There was heterogeneous contrast enhancement of the spleen (differentials including early post‐contrast, splenitis, extramedullary haematopoiesis or nodular hyperplasia). There was moderate enlargement of the left hepatic, medial iliac, internal iliac, sternal and axillary lymph nodes, which were described as most likely reactive. There were no intraparenchymal cysts observed. Compared to ultrasound, where the observed peritoneal cysts were innumerable, only the largest cysts were observed by CT scan.

### 4.2 Exploratory Laparotomy

**FIGURE 3:**
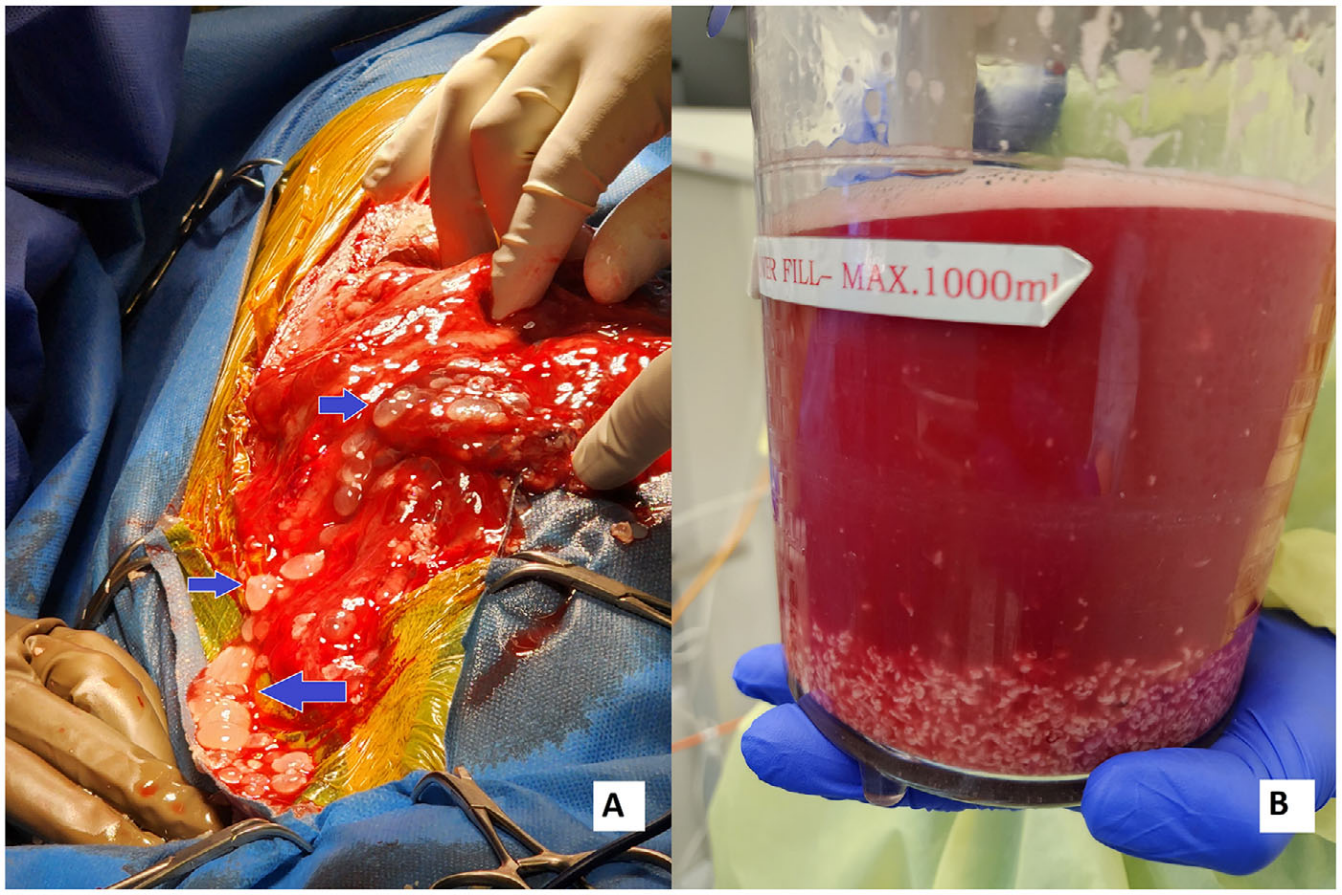
Exploratory laparotomy images showing numerous variably sized pale white‐yellow fluid‐filled parasitic cysts and floccular material (plerocercoids, blue arrows) pouring out of the abdominal cavity (A) and suspended within a large volume of serosanguinous peritoneal effusion (B).

**FIGURE 4:**
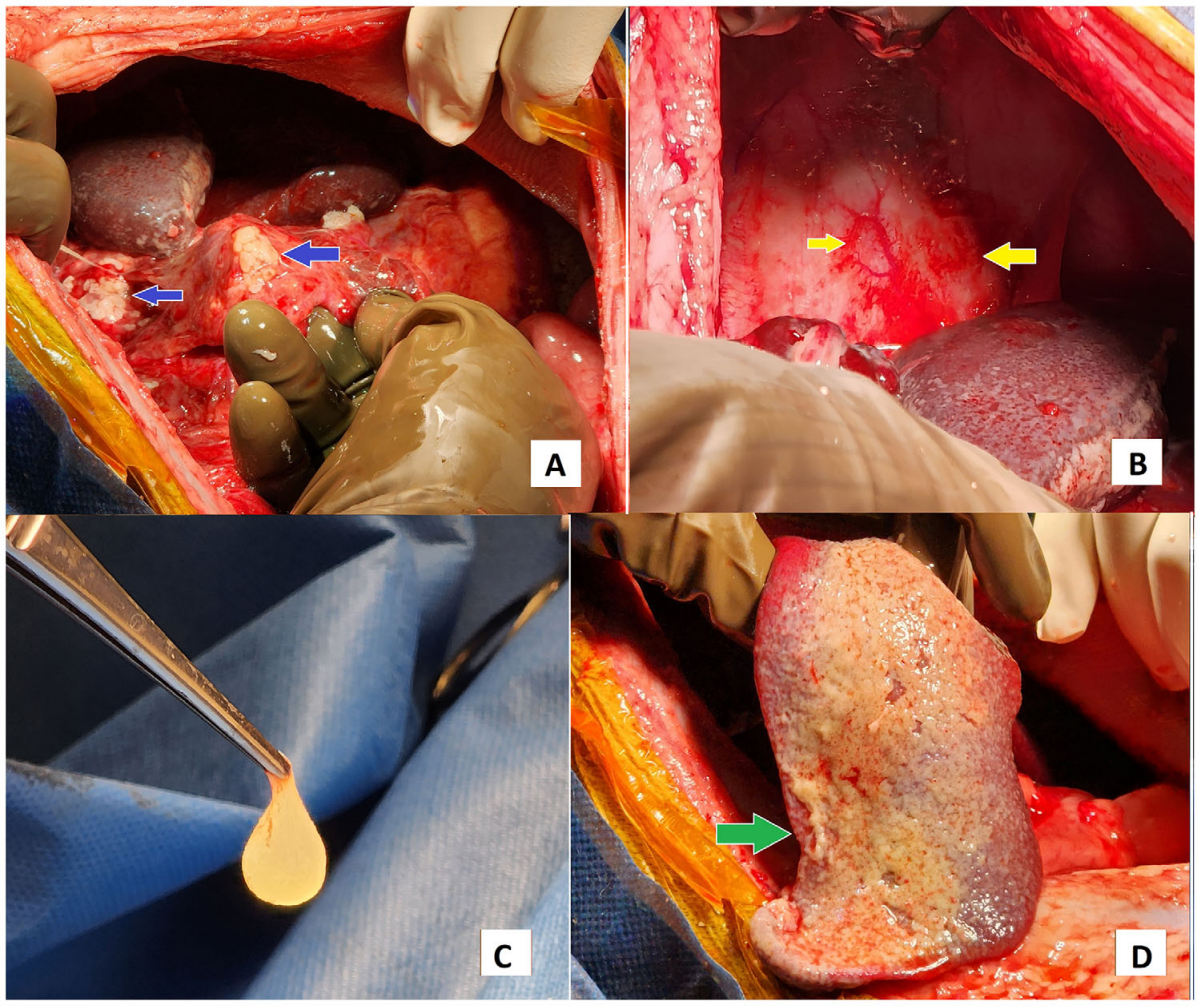
Exploratory laparotomy images showing diffuse bright red discolouration and plerocercoids forming soft white plaques (blue arrows) on the mesentery and omentum (A) and parietal peritoneum (B), with multifocal dark red areas (petechiae, yellow arrows). An individual plerocercoid cyst (C) and fibrous membrane on the surface of the spleen (D), green arrow.

An exploratory laparotomy was performed, suctioning approximately 3.5 L of serosanguinous turbid fluid with hundreds of free‐floating cystic structures from the peritoneal cavity (Figure 3, Supplement Video 2 and 3). The cysts were round, pale white to yellow with fluid and admixed floccular material within and measured between 1 to 12 mm in diameter. The peritoneal surface of the diaphragm, mesentery and omentum had dozens of round, dark red areas, measuring from 0.5 to 1 mm in diameter (presumed petechiae), and diffuse, bright red discolourations (erythema), with dozens of white, soft plaques on the serosal surface (Figure 4). The liver margins were slightly rounded, and the peripheral surfaces of the left medial and quadrate lobes were irregular to nodular. The surfaces of the splenic capsule and the hepatic diaphragmatic capsule had a fibrinous membrane. The parietal peritoneum was thickened and mildly irregular. The abdomen was lavaged with 12 L of warmed sterile isotonic saline and closed routinely. The peritoneal fluid was collected and sent for further examination. Post‐surgery, the patient was 3.7 kg lighter.

The peritoneal fluid had a high nucleated cell count (10.7 × 10^9^/L; RI: <1.5 × 10^9^/L), consisting of moderate numbers of macrophages, lymphocytes and neutrophils, and a protein of 18 g/L; RI <25 g/l. Within the fluid were acoelomic cestode tissues, with amorphous degenerate debris and calcareous corpuscles, consistent with peritoneal cestodiasis. The bacterial culture was negative.

### 4.3 Histopathology

**FIGURE 5:**
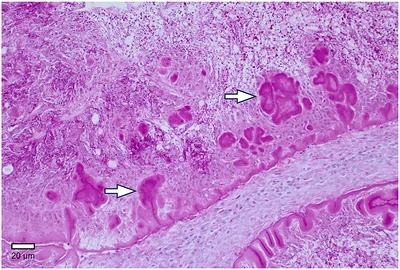
The tegument of the parasite frequently invaginates into the parenchyma (white arrows), Periodic Acid Schiff stain (200X magnification).

The omental biopsy noted severe chronic diffuse eosinophilic granulomatous peritonitis and eosinophilic and neutrophilic omental steatitis, with intralesional solid and/or fluid‐filled larval cestodes. Occasionally, granulomas surrounded lakes with eosinophilic amorphous material admixed with clear acicular cholesterol clefts. Histologically, the cestodes appeared solid segmented, covered by an eosinophilic tegument and an internal parenchyma with scattered basophilic round to ovoid calcareous corpuscles and excretory canals, and lacked a coelomic cavity. Frequently, the tegument had tubular invaginations, which extended into the parenchyma. (Figure 5).

### 4.4 Genetic Characterisation of Cestode Vesicles

Vesicle structures recovered from the peritoneal cavity during exploratory laparotomy (*n =* 8) were characterised genetically by targeting a region of the mitochondrial cytochrome *c* oxidase I (COX1) as described previously (Hu et al. 2005). Genomic DNA was extracted using a DNeasy Blood and Tissue Kit (Qiagen, USA) following the standard manufacturer protocol. PCR was carried out in a 25 µL volume containing 10 mM Tris‐HCl (pH 8.4), 50 mM KCl (Promega, USA), 3.5 mM of MgCl_2_, 200 µM of each deoxynucleotide triphosphate, 50 pmol of each primer (JB3, 5’‐TTTTTTGGGCATCCTGAGGTTTAT‐3’ and JB4.5, 5’—TAAAGAAAGAACATAATGAAAATG – 3’) and 1 U of Go*Taq* polymerase (Promega) under the following cycling conditions: 94°C for 5 min (initial denaturation); 35 cycles of 94°C for 30 s (denaturation), 52°C for 30 s (annealing) and 72°C for 30 s (extension), followed by a final extension at 72°C for 5 min. For each PCR, negative (no‐DNA) and positive (*Taenia hydatigena*) controls were included. No amplification was detected in any of the negative control reactions during this study. Amplicons (5 µL) were examined on 1.5% agarose gels stained with Gel Red Nucleic Acid Stain (Biotium, Inc. Hayward, CA, USA). Gels were examined using a ChemiDoc MP Imaging System (BioRad, Hercules, CA, USA). The COX1 amplicons produced from vesicle DNA were consistent in size (∼418 bp) with that of the *T. hydatigena* positive control sample, and no size variation was detected on agarose gels among any of the eight amplicons. PCR amplicons were purified using a FavorPrep^TM^ GEL/PCR Purification Kit (Favorgen Biotech Corp., Taiwan) prior to DNA Sanger sequencing using the primers JB3 and JB4.5 in separate reactions. The quality of each sequence was appraised using the program Geneious Prime (Biomatters Ltd., Auckland, New Zealand). The DNA sequences determined herein have been submitted to the GenBank database (GenBank accession number PX381206). BLAST analyses of the 418 bp DNA sequence obtained for each of the vesicle samples showed identity to that of a specimen of *Spirometra mansoni* (GenBank accession number MT131821) from a lowland copperhead snake collected from Victoria, Australia (Zhu et al. 2002).

### 4.5 Diagnosis

Peritoneal proliferative sparganosis due to *S. mansoni*.

## 5 Post‐Operative Observations

The patient recovered well post‐surgery and was discharged the following day, starting oral medications as labelled in the Appendix Table A1. One week post‐operatively, the patient had lost 1 kg but was otherwise well. In the second week, the owner had noted a reduction in appetite, softer stools and a single vomit. A focused abdominal ultrasound was performed noting moderate echogenic peritoneal effusion, hyperechoic mesentery, and small intestinal corrugation. The changes were consistent with non‐specific gastroenteritis and attributed to the medications; however an ongoing inflammatory peritonitis secondary to the sparganosis was also considered. Three weeks post‐operatively, the patient presented with lethargy, hyporexia, ongoing vomiting and urinary incontinence, with a further 2 kg weight loss. Examination noted pale mucous membranes, a dull demeanour, tachycardia, and a mid‐cranial firm mass‐like structure on abdominal palpation. The patient was re‐admitted for analgesia and diagnostics.

### 5.1 Haematology and Biochemistry

Haematological and biochemical analyses revealed an inflammatory leukogram (24.4 × 10^9^/L; RI: 6–17 × 10^9^/L) with moderate neutrophilia (19.9 × 10^9^/L; RI: 3–11.5 × 10^9^/L) with left shift and toxic change, monocytosis (2.5 × 10^9^/L; RI: 0.2‐1.4 × 10^9^/L), a mild hypokalaemia (3.7 mmol/L; RI: 3.8‐5.6 mmol/L), mild hypercholesterolaemia (8.3 mmol/L; RI: 3.9‐7.8 mmol/L), increased ALP (468 U/L; RI: 0–170 U/L), increased amylase (2106 U/L; RI: 180–1200 U/L), increased lipase (528 U/L; RI: 0–395 U/L), and borderline hypoalbuminaemia (30 g/L; RI: 31–44 g/L). Free catch urine was noted as highly concentrated (USG 1.050) and aciduric urine (pH 5), with mild dipstick bilirubinuria (2+), proteinuria (1+) without evidence of infection.

### 5.2 Abdominal Ultrasound

Ultrasound showed mild to moderate volumes of echogenic peritoneal effusion, scattered variably sized anechoic cysts, and extensive hyperechoic mesenteric strands connecting the small intestinal loops to the colon and cranial bladder wall (peritonitis and adhesions). The stomach was marked distended with anechoic fluid, and the small and large intestinal walls were variably thickened, diffusely corrugated, and plicated, with the observed bunching together centrally. The cranial bladder wall was markedly thickened, measuring 7.8 mm; RI: 1.4‐1.6 mm (Geisse et al. 1997). The liver was diffusely hypoechoic; the gall bladder contained echogenic sludge, with thickened hyperechoic irregular walls measuring 2.6 mm; RI 1.1‐1.4 mm (Martinez et al. 2023). Vomiting, hyporexia, weight loss, urinary incontinence, and palpable abdominal mass were attributed to the marked peritonitis and mesenteric adhesions, acting as a physical obstruction of the gastrointestinal tract, with secondary pancreatitis, cholestasis, cystitis, and borderline hypoalbuminaemia. Aciduria likely reflected hyperlactataemia (not tested) due to other causes of acidosis not observed. Owing to a poor prognosis, the animal was euthanised, and a postmortem was performed.

## 6 Postmortem

**FIGURE 6:**
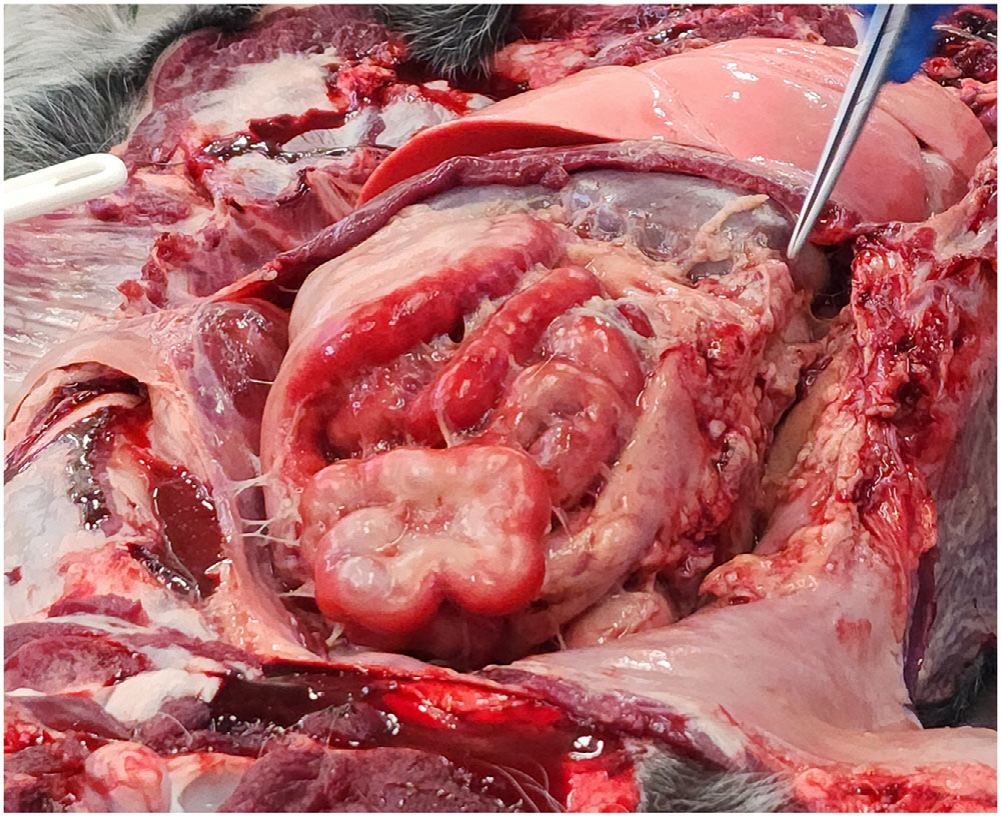
Postmortem image showing extensive firm pale‐tan stringy fibrinous adhesions, indicated by the silver forceps, connecting the ventral peritoneal body wall, intestinal serosa, omentum and liver, and dark red transverse plications affecting more than 70% of the entire small intestinal serosal surface.

Gross examination found an extensive coagulum of firm, pale tan, and stringy adhesions, encompassing the ventral peritoneal body wall, intestinal serosa, omentum, liver, and diaphragm (Figure 6), with approximately 240 mL of red, thin peritoneal fluid. The intestinal serosal surface was diffusely bright red, with dark red transverse plications affecting more than 70% of the entire small intestinal serosal surface. The inter‐serosal adhesions had dozens of round cystic spaces containing pale tan fluid, measuring 5 to 15 mm in diameter, admixed with pale cream, stringy material measuring 1 × 1 × 3.3 mm (cestode organisms). The cranial mediastinal lymph nodes were enlarged.

**FIGURE 7:**
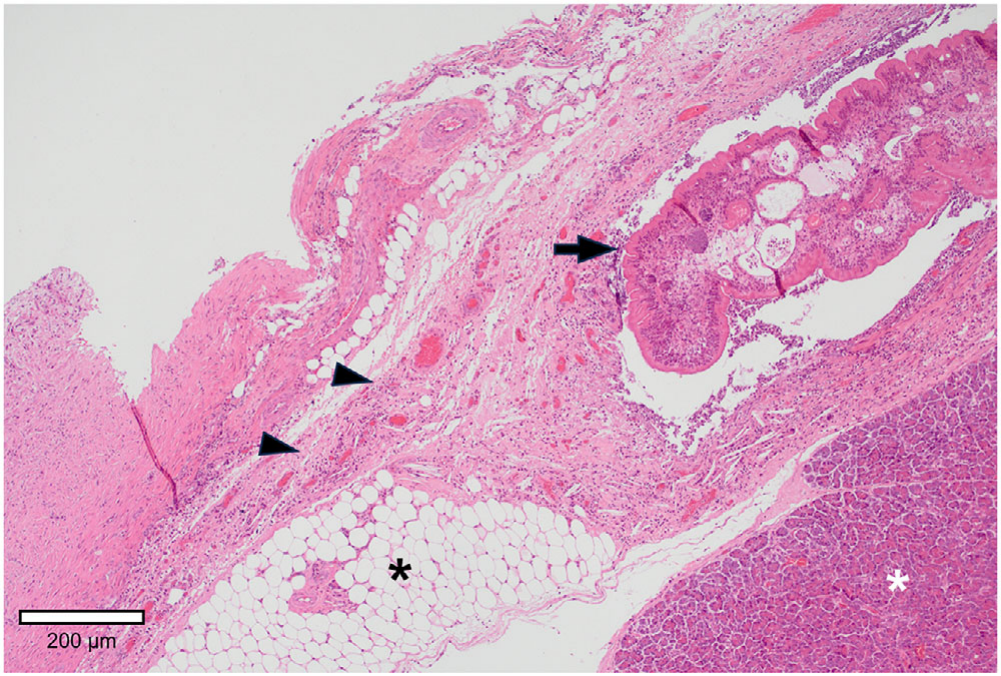
Postmortem peritoneum: A cestode parasite (black arrow), adjacent to the pancreas (white asterisk), is surrounded by granulomatous inflammation and fibrosis (black arrowheads) of peritoneal adipose tissue (black asterisk). Haematoxylin and eosin stain (40X magnification).

On histopathology, the peritoneum and capsular surfaces of the pancreas, liver and spleen the serosal surfaces of the stomach, diaphragm, mesentery and omentum had diffuse, severe granulomatous peritonitis and extensive fibrosis. Adjacent to the pancreatic capsule, there was an encapsulated larval cestode (Figure 7), measuring approximately 2 × 0.7 mm, consisting of an external, undulating, eosinophilic tegument and a parenchyma containing scattered basophilic concentric structures (calcareous corpuscles). The parenchyma contained dilations of clear space admixed with occasional degenerate neutrophils and occasional eosinophils, and necrotic debris (degenerate cestode parenchyma). The hepatic parenchyma had areas of hepatocellular atrophy, with biliary hyperplasia and cholestasis. The mediastinal lymph nodes had diffused moderate‐severe lymphadenitis.

## 7 Discussion

This case report documents a detailed diagnostic picture of peritoneal proliferative sparganosis in an Australian dog, describing the findings on ultrasound, computed tomography, exploratory laparotomy, histopathology, molecular testing and postmortem examination, and adding to the limited literature describing this topic.

To the authors’ knowledge, this is the first case report to describe the imaging features of proliferative sparganosis, using ultrasound and computed tomography. While the latter was able to identify the marked peritoneal effusion and larger nodules, it was less sensitive at identifying the small peritoneal cysts, as seen sonographically. By ultrasound, the innumerable small anechoic cysts were easily identified, ranging in size from 2 to 18 mm, with a thin hyperechoic wall, floating free within the peritoneal cavity and surrounded by large volumes of peritoneal effusion. While ultrasound interpretation relies on user skill and experience, the numerous peritoneal cysts could be identified on a point‐of‐care ultrasound, and increased awareness of this condition could lead to parasitic infections being included on the initial differential list. Ultrasound has been used to document other parasitic infections (Corda et al. 2022), including *Echinococcus* spp. and *Mesocestoides* spp., with the latter causing peritoneal larval cestodiasis and displaying similar findings on ultrasound and exploratory laparotomy as observed in this case. *Mesocestoides* spp., however, have not been reported in Australia (CDC 2024). The sonographic features seen with larval cestodiasis include anechoic fluid‐filled cystic structures measuring between 1–20 mm, and volumes of particulate abdominal effusion (Venco et al. 2005; Carta et al. 2021), and exploratory laparotomy findings include serohaemorrhagic peritoneal effusion, free and partly adhered cystic structures, fibrinous adhesions (Carta et al. 2021) and severe peritonitis (Aypak et al. 2012). Therefore, outside of Australia, both *Spirometra* and *Mesocestoides* spp. should also be considered as differentials for innumerable anechoic peritoneal cysts identified on ultrasound or exploratory laparotomy.

Proliferative sparganosis is very rarely reported, with only two published Australian articles describing this in canines (Simpson et al. 2012; Beveridge et al. 1998). One report describes numerous plerocercoids scattered throughout the thoracic cavity of an older Labrador, while the other describes five cases of peritoneal proliferative sparganosis, three of which were noted incidentally. The larval cestodes were tentatively diagnosed as *Spirometra* spp. by their appearance under electron microscopy and three confirmed to be *S. erinacei* (now termed *S. mansoni*) by molecular testing (Simpson et al. 2012; Beveridge et al. 1998). One case responded to treatment, while the others didn't or were either euthanised due to a poor prognosis, which is observed across the literature (Simpson et al. 2012; Semenova et al. 2025; Drake et al. 2008; Stief and Enge 2011) and this case report.

*Spirometra spp*. by undergone a number of taxonomic name changes over the years (Yamasaki et al. 2021; Kuchta et al. 2021), and until recently, all articles describing *Spirometra* in Australia were described as *S. erinacei*, synonymous with *S. erinaceieuropaei*. However, a recent molecular study determined that the species present in Australia is *S. mansoni* (Yamasaki et al. 2021). In addition, the varied use of parasitic terminology has led to challenges comparing the literature; for example, the term plerocercoid appears interchangeably with the terms spargana, sparganum and metacestode (larval stage of cestode).

Globally, canine proliferative sparganosis is also rarely reported, with two articles (Drake et al. 2008; Stief and Enge 2011) describing a closely related but genetically different (Miyadera et al. 2001; Kikuchi et al. 2021) species named *Sparganum proliferum* as responsible for causing proliferative sparganosis. In both cases, the dogs also presented with serosanguinous effusion, proliferative peritonitis, fibrosis, adhesions and numerous parasitic cysts (Drake et al. 2008; Stief and Enge 2011), with a diagnosis of *S. proliferum* larvae by their morphologic features (identical to *Spirometra* spp.). Molecular testing was not performed in these cases, and it was historically thought that *Spirometra* spp. larvae do not replicate asexually or form cysts (Stief and Enge 2011), leading to a diagnosis of *S. proliferum*. This is conflicting with the data presented in this case report, and other recent studies (Miller et al. forthcoming; Semenova et al. 2025), which have shown that *Spirometra* plerocercoids can proliferate asexually. Asexual replication of *Spirometra* larvae was also recently observed in an aberrant sparganosis case in a cat from Japan (Tokiwa et al. 2024), noting multiple asexual *S. mansoni* larvae within a lesion, suggestive of proliferative sparganosis. Newer studies have recommended molecular testing as the most reliable tool to identify *Spirometra* spp., and have described using morphologic criteria to identify *Spirometra* larvae as unsuitable and unreliable (Yamasaki et al. 2021; Kuchta et al. 2021). Various forms of sparganosis (*Spirometra* or *Sparganum*) have also rarely been reported in cats from Japan, the USA and Vietnam, with three and two cases observing parasites in subcutaneous tissues and the abdominal cavity, respectively (Tokiwa et al. 2024; Buergelt et al. 1984, Woldemeskel 2014; Nguyen et al. 2024). The limited number of cases and various presentations of sparganosis have led to the ongoing difficulty in comparing exposure and possible treatment options.

In humans, over 1,600 sparganosis cases have been reported globally (Liu et al. 2015), with subcutaneous (non‐proliferative) sparganosis being the most common form (Lu et al. 2014; Kim et al. 2018). Proliferative sparganosis in humans is rarely described and is also associated with a high mortality (Lescano and Zunt 2013; Kikuchi and Maruyama 2020), and therefore recognition of this condition in animals has important zoonotic implications.

This condition posed a diagnostic challenge, with a non‐specific history, examination, and haematology and biochemical findings and an absence of parasitic material identified on the initial ultrasound‐guided abdominocentesis sample, leading to a broad differential list. The subsequent peritoneal effusion sample obtained during the exploratory laparotomy contained numerous acoelomic cestode tissue and calcareous corpuscles, which narrowed the differentials to a parasitic condition. It is likely that the rupturing of the plerocercoid cysts during the laparotomy led to a greater number of free cestode tissues present within the peritoneal effusion sample, in addition to a larger sample volume collected compared to that obtained initially. Histopathology allowed further narrowing of the differentials, documenting severe granulomatous peritonitis with intralesional solid type larval cestodes, which was consistent with several other case reports of sparganosis in domestic animals (Beveridge et al. 1998; Drake et al. 2008; Stief and Enge 2011). The omental biopsy also demonstrated cestode profiles with a unique tegument invagination forming tubules that extend into the parenchyma, which has been previously documented (Beveridge et al. 1998), and its purpose is currently unknown. Further investigation is needed to determine if this descriptive feature is specific to *Spirometra* spp.; if so, histological assessment of tissue cestode sections may aid in early detection of this disease.

The prevalence of *Spirometra* in its definitive hosts has not been evaluated in Victoria. However, elsewhere *Spirometra* was found in 36% of the wild dog population's gastrointestinal tracts in New South Wales and Queensland (Harriott et al. 2019) and 5.4% of red foxes in Western Australia (Dybing et al. 2013). This is important to note, as the wild dog population acts as a reservoir for the adult worm and allows the continuation of the *Spirometra* life cycle, resulting in continued environmental, intermediate host, and paratenic host exposure to the larval form of the parasite and risk of sparganosis. In the presented case, the exposure source is unknown, with speculation of frogs or tadpoles acting as a potential infection source given the nearby wetlands; however, molecular testing confirmed the patients sample belongs to the non‐amphibian genotype, consistent with other samples obtained from the dog, fox, cat and tiger snake (Zhu et al. 2002). As *S. mansoni* requires multiple hosts to complete its life cycle, direct animal‐to‐animal transmission does not occur. However, the risk to other pets and wildlife in Victoria is contingent on the local prevalence of the parasite within definitive hosts (e.g., wild canids and client‐owned dogs and cats), intermediate hosts (e.g., copepods), and paratenic hosts (e.g., frogs, tadpoles, and snakes), as well as the degree of environmental contamination of local water bodies with parasite eggs. Further research is needed to assess the prevalence of *Spirometra* spp. in Victoria in its definitive hosts, intermediate hosts and local environment to better understand the risk to other pets and the risk of zoonosis locally. In terms of control and prevention of sparganosis, pet owners should be advised to prevent their animals from scavenging or hunting native fauna and to ensure regular anthelmintic treatment as part of routine parasite control programmes. Veterinarians can play a key role in surveillance by reporting confirmed cases and educating pet owners about the risks associated with wildlife contact.

As an inherent limitation of a single case report, the clinical presentation, imaging findings and disease progression described here may not be representative of all cases of proliferative sparganosis. Additional studies involving a larger number of animals are required to better characterise the spectrum of clinical and diagnostic findings associated with this condition. In the present case, advanced disease was characterised by progressive abdominal distension, weight changes, serosanguinous peritoneal effusion, extensive granulomatous peritonitis and marked intra‐abdominal adhesions. Ultrasonography proved particularly informative, revealing numerous free‐floating anechoic cystic structures within the peritoneal cavity, suspended in a large volume of echogenic peritoneal effusion. Computed tomography demonstrated marked abdominal effusion and nodular peritoneal lesions consistent with granulomatous inflammation. Given the rarity of proliferative sparganosis and the limited literature describing its imaging features, the findings presented in this report may assist veterinarians in recognising and investigating similar cases in the future.

## 8 Conclusions

This article presents a detailed diagnostic picture of peritoneal proliferative sparganosis due to *S. mansoni* in an Australian dog. It is the first of its kind to detail the findings on ultrasound and CT, as well as exploratory laparotomy and postmortem. Proliferative sparganosis is a rare and often fatal parasitic disease, responding poorly to anti‐parasitic treatment. It has a complex lifecycle and ever‐changing taxonomic challenges. Molecular genetic testing is required for a definitive diagnosis. It underscores the importance of considering this rare zoonotic disease as a differential for abdominal distension in dogs.

## Author Contributions

**Stephanie Middlemast**: conceptualisation, investigation, resources, writing – original draft preparation, writing – review and editing, visualisation. **Nicholas Lim**: conceptualisation, resources, writing – original draft preparation, writing – review and editing. **Charles G. Gauci**: writing – review and editing. **Abdul Jabbar**: conceptualisation, resources, writing – original draft preparation, writing – review and editing.

## Funding

The authors have nothing to report.

## Ethics Statement

Due to the retrospective nature of the case report, an ethics committee was not necessary.

## Consent

Written informed consent was obtained from the owner of the animal.

## Conflicts of Interest

The authors declare no conflicts of interest.

## Supporting information




**Video 1**: Ultrasound clip highlighting the innumerable variably sized anechoic cysts suspended in a large volume of peritoneal effusion, interlaced with strands of hyperechoic mesentery and small intestinal segments.

**Video 2**: Numerous variably sized pale white‐yellow fluid‐filled parasitic cysts (plerocercoids) flowing out from the exploratory laparotomy incision.

**Video 3**: *Spirometra mansoni* plerocercoid larvae suspended in saline observed moving and contracting under a stereomicroscope (40x magnification).

## Data Availability

Data sharing not applicable to this article as no datasets were generated or analysed during the current study.

## APPENDIX

### 1

**TABLE A1:** Post operative oral medication with respective dose, frequency, and duration of treatment. SID once daily, BID twice daily, TID three times daily.

Day	Medication	Dose	Frequency	Duration
Day 1	Meloxicam	0.1 mg/kg	SID	7 days
Day 1	Amoxicillin clavulanic acid	22 mg/kg	BID	7 days
Day 2	Fenbendazole	50 mg/kg	BID	21 days
Day 2	Praziquantel	25 mg/kg	SID	2 days
Day 4	Praziquantel	13 mg/kg	SID	19 days
Day 10	Ondansetron	0.3 mg/kg	BID‐TID	As needed
Day 10	Prokolin	5 mL	BID	3 days
Day 14	Nitazoxanide	90 mg/kg	SID	Once
Day 15	Ondansetron	0.5 mg/kg	BID‐TID	As needed
